# Baseline Sleep Literacy, Habits, and Perceptions Among Primary School Children: Foundations for a MAIEC-Guided Community Intervention in Sleep and Mental Health

**DOI:** 10.3390/healthcare14030390

**Published:** 2026-02-03

**Authors:** Pedro Melo, Joana Bastos, Paula Moreira, Carlos Pinto, Vanessa Monteiro, Jóni Madureira, Bárbara Ferreira, Filipe Rodrigues-Pires, Teresa Martins, Ana Paula Cantante

**Affiliations:** 1 School of Nursing, University of Porto, 4200-072 Porto, Portugal; 2 RISE-Health, MAIEC Lab, School of Nursing, University of Porto, 4200-072 Porto, Portugal

**Keywords:** child health, sleep, mental health, MAIEC, community empowerment, school-based intervention

## Abstract

**Highlights:**

- Baseline assessment of third-grade children revealed heterogeneous Sleep Habits and variable levels of sleep-hygiene knowledge across both school communities.
- Three composite indices were developed—Sleep Literacy (SLI), Sleep Habits (SHI), and Sleep Symptoms/Perceptions (SSPI)—to capture modifiable determinants of sleep linked to mental-health vulnerability.
- Children in the experimental community showed higher baseline scores across all indices, with the difference in Sleep Habits (SHI) approaching statistical significance and moderate effect sizes observed.
- No sex differences were observed in Sleep Literacy, Habits, or sleep-related perceptions, supporting the neutrality of sex as a determinant in this sample.
- These findings represent only the child-level baseline data from the broader quasi-experimental MAIEC-guided study.
- The quasi-experimental intervention is already underway, and the level of community empowerment has been assessed in both school communities as part of the MAIEC diagnostic process.
- The evaluation of the Community Process, using the MAIEC questionnaire, will be presented in a subsequent article focused on community-level assessment, diagnosis, and prediction of intervention pathways for the experimental community as the unit of care.
- The results reinforce the importance of early, school-based sleep-health promotion and provide a structured diagnostic foundation for the MAIEC-guided community-empowerment intervention.

**Abstract:**

Sleep plays a fundamental role in children’s cognitive, emotional, and behavioral development, and inadequate sleep hygiene is increasingly recognized as a modifiable risk factor for mental-health vulnerability. **Background/Objectives:** This article presents the child level baseline component of a broader quasi experimental study grounded in the Community Assessment, Intervention and Empowerment Model (MAIEC). The aim was to characterize Sleep Habits, sleep-hygiene knowledge, and sleep-related perceptions among third-grade children from two primary-school communities in northern Portugal, prior to the implementation of a MAIEC-based community intervention. **Methods:** Regarding the reporting, only the baseline child level assessment of the study presented in the manuscript, a structured questionnaire, integrating the Portuguese version of the Children’s Sleep Habits Questionnaire (CSHQ PT) and questions about Sleep Habits and sleep-hygiene literacy were administered to 40 children. Three composite indices were computed: Sleep Literacy (SLI), Sleep Habits (SHI), and Sleep Symptoms and Perceptions (SSPI). **Results:** Baseline results revealed heterogeneous Sleep Habits and variable sleep-hygiene knowledge. Children in the experimental community demonstrated higher scores across all indices, with the difference in Sleep Habits (SHI) approaching statistical significance (*p* = 0.052) and moderate effect sizes observed overall. No sex-based differences were found. **Conclusions:** These findings identify modifiable behavioral and knowledge-related sleep determinants linked to emotional regulation and mental-health risk. This article reports baseline child-level data from a broader MAIEC-guided study, with the quasi-experimental intervention currently underway and community empowerment already assessed. Future work will present the evaluation of community processes. Overall, these baseline results provide a structured diagnostic foundation for a MAIEC-based community intervention aimed at promoting healthy sleep and strengthening mental-health resilience in primary-school settings.

## 1. Introduction

Sleep is a fundamental determinant of children’s physical, cognitive, and socio-emotional development. Inadequate sleep duration and quality are associated with irritability, emotional dysregulation, attentional problems, and worse learning outcomes throughout childhood and adolescence. Framing sleep health as a pillar of mental-health promotion is therefore essential for school-aged children, where early, preventive, and school-wide strategies can have population-level impact [1,2,3,4,5,6]. 

Sleep difficulties are common in primary-school children, with international studies reporting high rates of insufficient sleep, irregular bedtimes, and difficulty initiating sleep in children aged 6–10 years. Such disruptions are consistently linked to poorer attention, reduced academic performance, and greater emotional dysregulation. These findings reinforce the need for early school-based assessment and intervention [1,3,4,5].

Schools are privileged settings for health promotion because they provide universal, stable access to children and families. Baseline characterization of Sleep Literacy, Habits, and sleep-related symptoms is a necessary diagnostic step to tailor interventions, identify modifiable determinants, and monitor change over time within a public-health framework [2].

This project is grounded in the Community Assessment, Intervention and Empowerment Model (MAIEC), a prescriptive middle-range nursing theory that positions the community as the unit of care and structures assessment, diagnosis, intervention, and evaluation [7,8,9,10]. Within this broader quasi-experimental study, the present article reports only the child-level baseline assessment conducted in two public primary schools in northern Portugal.

We selected the Portuguese version of the Children’s Sleep Habits Questionnaire (CSHQ-PT) because it is widely used internationally to assess sleep behaviors and problems in school-age children and has demonstrated validity and reliability in Portuguese samples [11,12]. Its content structure captures modifiable routines and symptoms that are directly actionable in school-based programs and aligned with the MAIEC focus on community-level determinants.

We aimed (1) to characterize baseline Sleep Literacy, Sleep Habits, and sleep-related symptoms/perceptions among third-grade children from two school communities; and (2) to identify modifiable behavioral and knowledge determinants relevant to subsequent MAIEC-guided community intervention.

Baseline data plays an essential standalone role in community-focused public-health research because they delineate the initial distribution of modifiable determinants before any external influence occurs. Publishing these data provides a transparent diagnostic foundation for future intervention effects and supports replicability across school communities.

## 2. Materials and Methods

This article presents only the baseline component of a broader quasi-experimental before–after study with a non-randomized control group conducted in two public primary schools.

### 2.1. Study Design

Quasi-experimental design with two naturally existing school communities: one designated as the experimental community where the MAIEC-based intervention will be implemented, and one control community without intervention during the study period. No individual randomization occurred; allocation operated at the school level.

### 2.2. Setting and Participants

The schools are integrated in the same Local Health Unit served by the school health nursing team. We used non-probability convenience sampling, including all third-grade students with parental consent and child assent. Group allocation reflected school membership (cluster allocation by school); no crossover occurred.

### 2.3. Measures

The construction of the three indices (SLI, SHI, SSPI) followed content-mapping procedures aligned with sleep-hygiene literature. Items included were selected based on conceptual coverage of key sleep determinants, age-appropriateness, and alignment with MAIEC diagnostic dimensions.

The newly developed items (Sleep Literacy and perceptions) underwent expert review by two sleep-health researchers and two school health nurses to ensure clarity and developmental adequacy. Although no formal pilot test was conducted, cognitive interviewing was embedded in classroom administration to verify comprehension.

We acknowledge limitations inherent to adapted and newly developed measures in young children, including concrete reasoning, limited introspective ability, and potential overestimation of positive behaviors. These limitations are addressed through index-level aggregation and triangulation with MAIEC community data in the larger study.

### 2.4. Procedures and Timing

Baseline data were collected between September and October 2025 by the school health nurses using standardized procedures in classroom settings. This manuscript reports exclusively the baseline assessment. Post-intervention assessments are planned for the subsequent school term within one year, in October 2026.

### 2.5. Variables and Index Construction

Three composite indices were constructed to summarize key domains: (i) Sleep Literacy Index (SLI), proportion of correct responses across 13 true/false items on sleep-hygiene knowledge (0–1, higher is better); (ii) Sleep Habits Index (SHI), mean of four items on bedtime routines, sleep onset, and schedule consistency (1–3, higher is better); and (iii) Sleep Symptoms and Perceptions Index (SSPI), mean of eight items reflecting perceived sleep quality and symptoms (1–3, higher is better).

### 2.6. Statistical Analysis

Descriptive statistics and compared groups (experimental vs. control; girls vs. boys) were computed using two-tailed independent samples *t*-tests. We examined normality via Shapiro–Wilk tests and Q–Q plots and assessed homogeneity of variances with Levene’s test. Given the approximately balanced group sizes and the robustness of the *t*-test to mild deviations, parametric tests were retained. *p*-values (α = 0.05) were reported alongside effect sizes (Cohen’s d) to emphasize practical significance. Analyses were conducted in jamovi (v2.7) [13].

## 3. Results

The descriptive data (Table 1, Table 2, Table 3 and Table 4) reveal a homogeneous age distribution and balanced sex composition. Narrative redundancy has been reduced to emphasize key trends only: Sleep Literacy showed the greatest dispersion, whereas Sleep Habits and perceptions were relatively uniform across children.

The predominance of eight-year-old children indicates a homogeneous age profile, strengthening the internal consistency of the baseline assessment.

Group sizes were comparable across the two communities, supporting between-group comparisons at baseline.

The sex distribution was approximately balanced; therefore, sex-based analyses were not confounded by unequal group sizes.

Descriptive statistics for SLI, SHI, and SSPI are summarized in Table 4; variability across children was greater for knowledge (SLI) than for behaviors or perceptions.

Knowledge showed wider dispersion than habits or perceptions, indicating heterogeneous understanding of sleep-hygiene concepts.

These findings highlight the coexistence of adequate sleep-related behaviors with gaps in conceptual knowledge, reinforcing the need for structured educational interventions.

Group comparisons (Table 5 and Table 6) showed small and non-significant differences across most indices. The moderate effect size for SHI (d ≈ 0.64) should be interpreted as exploratory rather than indicative of a substantive baseline distinction between communities.

Between-community comparisons (Table 5) suggest a moderate advantage for the experimental community in Sleep Habits (SHI; d ≈ 0.64) despite a *p*-value near the conventional threshold, whereas differences in SLI and SSPI were small and not statistically significant.

Although none of the comparisons reached conventional statistical significance (*p* < 0.05), the Sleep Habits Index (SHI) approached significance (*p* = 0.052), with a moderate effect size (d = 0.638) favoring the experimental community. The Sleep Literacy Index (SLI) showed a small to moderate effect size (d = 0.360), despite a non-significant *p*-value (*p* = 0.264), suggesting a potential practical difference. The Sleep Symptoms and Perceptions Index (SSPI) revealed a negligible and non-significant difference (*p* = 0.598, d = −0.169), with slightly lower scores in the experimental group.

Overall, baseline differences are modest and primarily behavioral, informing targets for subsequent intervention.

No sex differences were detected across indices (Table 6); effect sizes were negligible to small.

None of the comparisons reached statistical significance. The Sleep Literacy Index (SLI) showed a non-significant trend favoring females (*p* = 0.211), with a small to moderate effect size (d = 0.402), suggesting a potential practical difference. The Sleep Habits Index (SHI) revealed identical mean scores between sexes (*p* = 1.000, d = 0.000), indicating no observable difference. The Sleep Symptoms and Perceptions Index (SSPI) also showed no significant difference (*p* = 0.687), with a negligible effect size (d = 0.129).

These findings suggest that sex does not appear to be a determining factor in sleep-related knowledge, habits, or perceptions within this sample, reinforcing the neutrality of sex as a variable in the context of this study.

## 4. Discussion

This study’s objectives were to describe Sleep Literacy, Habits, and perceptions and to identify modifiable determinants among third-grade children from two public school communities. We observed heterogeneous sleep knowledge alongside generally moderate-to-positive habits and perceptions, a pattern consistent with the notion that behaviors can be acceptable even when conceptual understanding is uneven.

Between-community differences were small overall. The SHI showed a moderate effect size favoring the experimental community at baseline, whereas SLI and SSPI differences were small and non-significant. In keeping with good reporting practice, we emphasize the magnitude and direction of effects rather than *p*-values close to α, as near-threshold findings are inherently unstable in small samples. Near-significant results (e.g., SHI *p* = 0.052) are interpreted cautiously. In small samples, *p*-values near threshold provide limited inferential value; effect sizes should instead be treated as preliminary signals that warrant further investigation rather than evidence of community-level advantage.

For practice, these results point to concrete, modifiable targets—bedtime routines, sleep schedule consistency, and stimulus control—well-suited to a school-wide program. Within the MAIEC framework, these child-level data inform the community diagnosis and the design of empowerment-oriented strategies engaging children, families, and teachers.

Strengths include the real-world, school-based setting, standardized procedures, and alignment with a theory-driven community framework.

Limitations include the small sample, cluster (school-level) allocation without randomization, reliance on child self-report, and the cross-sectional nature of the present analysis. Assumption checks supported the use of *t*-tests; nonetheless, all baseline comparisons should be interpreted as exploratory. Individual health or socioeconomic variables could not be collected due to national ethical restrictions. Although small, the sample represents the full population of the two school communities, consistent with MAIEC community-level diagnostics.

The ongoing quasi-experimental study will report post-intervention changes at child and community levels, including the MAIEC Community Process evaluation, enabling an integrated diagnosis and prediction of intervention pathways.

## 5. Conclusions

In this baseline assessment of two primary-school communities, children displayed heterogeneous Sleep Literacy and modestly favorable Sleep Habits and perceptions. Baseline differences between communities were small, with a moderate advantage in Sleep Habits in the experimental setting.

These findings provide a focused diagnostic for a MAIEC-guided intervention that prioritizes consistent routines and sleep-hygiene education. Given the small sample and non-randomized allocation, results are preliminary; the forthcoming post-intervention analyses will determine whether community-level action translates into meaningful improvements in child sleep health and related mental-health resilience.

## Figures and Tables

**Table 1:** Age distribution of the sample.

Age	Frequency	Percentage
7	1	2.5%
8	35	87.5%
9	3	7.5%
10	1	2.5%
	40	100%

**Table 2:** Community distribution of the sample (control and experimental community).

Community	Frequency	Percentage
Control	22	55%
Experimental	18	45%
	40	100%

**Table 3:** Sex distribution of the sample.

Sex	Frequency	Percentage
Female	20	50%
Male	20	50%
	40	100%

**Table 4:** Descriptive statistics for the three composite indices: Sleep Literacy Index (SLI), Sleep Habits Index (SHI), and Sleep Symptoms and Perceptions Index (SSPI).

Index	N	Mean	Median	SD	Min	Max
SLI	40	0.660	0.615	0.202	0.308	1.310
SHI	40	2.020	2.000	0.362	1.250	2.500
SSPI	40	1.910	1.750	0.192	1.750	2.250

**Table 5:** Results of independent samples *t*-tests comparing control and experimental groups on sleep-related indices.

Index	Test Statistics	df	*p*-Value	Mean Difference	Standard Error of the Difference	Effect Size (Cohen’s d)
SLI	Student’s t	38	0.264	0.0726	0.0641	0.360
SHI	Student’s t	38	0.052	0.2222	0.1107	0.638
SSPI	Student’s t	38	0.598	−0.0328	0.0617	−0.169

**Note**: H_a_: μ Control ≠ μ Experimental.

**Table 6:** Independent samples *t*-test comparing female and male participants.

Index	Test Statistics	df	*p*-Value	Mean Difference	Standard Error of the Difference	Effect Size (Cohen’s d)
SLI	Student’s t	38	0.211	0.0808	0.0635	0.402
SHI	Student’s t	38	1.000	0.0000	0.1158	0.000
SSPI	Student’s t	38	0.687	0.0250	0.0615	0.129

**Note**: H_a_: μ Female ≠ μ Male.

## Data Availability

Data is unavailable due to privacy or ethical restrictions, in a data base at MAIEC Lab Office 365 professional account (based on University of Porto School of Nursing) with exclusive access to the research team.
